# Outcomes and risk factors with COVID-19 or influenza in hospitalized asthma patients

**DOI:** 10.1186/s12931-022-02265-6

**Published:** 2022-12-13

**Authors:** Axelle Dupont, Camille Couffignal, Camila Arias, Kankoe Salah, Mathilde Phillips-Houlbraq, Mathilde Le Brun, Camille Taillé

**Affiliations:** 1 Clinical Research, Biostatistics and Epidemiology Department, AP-HP Nord-Université Paris Cité, HUPNVS, Paris, France; 2 Université Paris Cité and Université Sorbonne Paris Nord, INSERM, IAME, Paris, France; 3 INSERM CIC-EC 1425, Hôpital Bichat Claude Bernard, Paris, France; 4 Respiratory Diseases Department, AP-HP Nord-Université Paris Cité, Hôpital Bichat Claude Bernard, Paris, France; 5 INSERM U1152, Paris, France

**Keywords:** Asthma, COVID-19, Influenza, Prognosis, Clinical Data Warehouses

## Abstract

**Background:**

At the time of the SARS-CoV-2 emergence, asthma patients were initially considered vulnerable because respiratory viruses, especially influenza, are associated with asthma exacerbations, increased risk of hospitalization and more severe disease course. We aimed to compare the asthma prevalence in patients hospitalized for COVID-19 or influenza and risk factors associated with poor prognosis with the diseases.

**Methods:**

This retrospective cohort study used the Paris university hospitals clinical data warehouse to identify adults hospitalized for COVID-19 (January to June 2020) or influenza (November 2017 to March 2018 for the 2017–2018 influenza period and November 2018 to March 2019 for the 2018–2019 period). Asthma patients were identified with J45 and J46 ICD-10 codes. Poor outcomes were defined as admission in intensive care or death.

**Results:**

Asthma prevalence was significantly higher among influenza than COVID-19 patients (n = 283/3 119, 9.1%, 95% CI [8.1–10.1] in 2017–2018 and n = 309/3 266, 9.5%, 95% CI [8.5–10.5] in 2018–2019 versus n = 402/9 009, 4.5%, 95% CI [4.0–4.9]). For asthma patients, 31% with COVID-19 were admitted to an intensive care unit versus 23% and 21% with influenza. Obesity was a risk factor for the 2017–2018 influenza period, smoking and heart failure for the 2018–2019 period. Among COVID-19 patients with asthma, smoking and obesity were risk factors for the severe form.

**Conclusions:**

In this study, patients with an asthma ICD-10 code were less represented among COVID-19 patients than among influenza-infected ones. However, outcomes were poorer for COVID-19 than influenza patients, both with asthma. These data highlight the importance of protective shields and vaccination against influenza and COVID-19 in this population.

**Supplementary Information:**

The online version contains supplementary material available at 10.1186/s12931-022-02265-6.

## Background

The new coronavirus SARS-CoV-2, first identified in December 2019, is responsible for coronavirus disease 19, the cause of a pandemic that has caused more than 5 million deaths from March 2020 to December 2021 [1]. Other respiratory viruses, specifically influenza viruses, are responsible for thousands of hospitalizations each year that varied among seasons, from 13,745 in 2013–2014 to 29,365 in 2016–2017 in France [2].

Asthma patients were first considered a vulnerable population for COVID-19 hospitalizations, from previous experience with other respiratory viruses [3, 4] and the susceptibility of the respiratory epithelium to viruses because of suppression of the interferon response by T helper 2 cell-type inflammation [5, 6]. This vulnerability can result in more severe infectious symptoms [7] or asthma exacerbations [5, 8, 9]. Indeed, during the H1N1 epidemic in 2009, asthma was one of the most common underlying medical conditions among hospitalized patients in the United States (25%) [10, 11] and the United Kingdom (25.3%) [12]. In the United States in 2019, asthma patients represented 22.3% of laboratory-confirmed hospitalizations for influenza [13]. Such patients are at increased risk of admission to an intensive care unit when infected with seasonal influenza virus [14] or were at increased risk during the H1N1 virus pandemic [10].

However, several countries have reported an unexpected under-representation of asthma patients among those hospitalized for COVID-19 [15, 16]. Reasons for this low hospitalization rate for COVID-19 among asthma patients are multiple: better protective shields, especially during lockdown restrictions [17, 18]; strong adherence to measures and treatment [19]; reduction of the transmission of other respiratory viruses and environmental exposure to allergens or pollutants [20, 21]; and changes in physical activity and/or diet [22]. The potential protective effect of inhaled corticosteroids on SARS-CoV-2 virus invasion through the bronchial epithelium has also been raised [23, 24]. However, some data suggest, as for influenza [25], that outcomes with COVID-19 are poorer for asthma patients than other patients, especially those using oral corticosteroids [26, 27].

Nevertheless, although asthma patients may be less frequently hospitalized for COVID-19 than expected, the associated morbidity is high: among French asthma patients hospitalized from March to April 2020 for COVID-19, 19.6% were admitted to an ICU [15]. Because of the risk of asthma exacerbations or poor outcome with these different respiratory viral infections, the search for specific risk factors is needed in order to adapt preventive measures.

The aim of our study was to use the institutional large Clinical Data Warehouse of Paris hospitals to compare asthma prevalence in patients hospitalized for COVID-19 or influenza and to describe and compare outcomes and risk factors for COVID-19 and influenza in a population of hospitalized asthma patients.

## Methods

### Study population

This retrospective cohort study used a large database, the institutional Clinical Data Warehouse of Greater Paris University Hospitals (Entrepôt de Données de Santé [EDS], https://eds.aphp.fr/). This data warehouse contains the electronic health records for all inpatients from the 39 greater-Paris-area university hospitals (Assistance Publique Hôpitaux de Paris [AP-HP]). The study was approved by the ethics committee of EDS (IRB00011591).

- Cohort of COVID-19 patients: we included adults (age > 18) with at least one SARS-CoV-2-positive PCR test result (nasal brushing test performed in one laboratory belonging to the AP-HP) between January 1 and June 30, 2020 and hospitalized for COVID-19 in one of the AP-HP hospitals. We excluded patients consulting in an emergency room not followed by a hospitalization. A hospital stay was considered to be related to COVID-19 if the PCR test was performed at a 15-day delay before the start of hospitalization or if a PCR test was positive during a hospital stay.
- Cohorts of influenza patients: we included adults hospitalized in one of the AP-HP hospitals with a discharge code of J09, J10, J11 [2] in the International Statistical Classification of Diseases, Tenth Revision (ICD-10) during the influenza periods studied. Dates of influenza periods were defined by the French Sentinelles network (French national system of clinical surveillance). Three seasons were considered, November 1 to March 31 in 2017–2018, 2018–2019 and 2019–2020.
- During the 2019–2020 season, hospital stay coding clearly differed from that for other years, with a higher number of J12 codes (see Additional file 1: Fig. S1). The SARS-CoV-2 virus likely co-circulated with other respiratory viruses in early 2020 [28], so comparisons between the 2019–2020 influenza and COVID-19 periods were difficult to interpret; therefore, we present results for this period in Additional file 1: Tables S4–S7.

### Definition of asthma and other comorbidities

Asthma patients were identified in electronic health records by using J45 (Asthma) and J46 (Status asthmaticus) ICD-10 codes. Patients with other comorbidities (ICD-10 codes in Additional file 1: Table S1) were identified by the same method, except for those with obesity and smoking (active smoking or smoking history), for which we also performed natural language processing (regular expressions for obesity or smoking) to identify these comorbidities in hospital discharge reports, in addition of ICD-10 codes search. Only comorbidities that were coded during a hospital stay could be retrieved. During the hospitalization for COVID-19 or influenza, admission to an ICU (Intensive Care unit) was searched among the list of wards where the patient had been hospitalized.

### Asthma treatments

Text mining based on regular expressions was used to identify inhaled corticosteroids prescriptions corresponding to Anatomical Therapeutic Chemical codes R03AK, R03AL, R03BA in COVID-19 or influenza hospital discharge reports.

### Statistical analysis

Asthma prevalence and Clopper–Pearson 95% confidence intervals were calculated. Fisher exact test was used to compare asthma prevalence between COVID-19 and influenza patients. We also compared characteristics of hospitalized asthmatic patients between each influenza period and COVID-19 patients by Wilcoxon rank sum test for quantitative data and Fisher exact test for categorical data. We compared asthmatic and non-asthmatic patients by disease with the same methodology.

For each disease, factors associated with poor outcome (ICU stay or death) were evaluated by univariate logistic regression adjusted for age, sex, obesity, diabetes, heart failure, atherosclerotic heart disease, smoking, stroke, chronic renal failure on dialysis. The linearity assumption for age was violated with analysis for COVID-19 patients, so we created a binary variable < 70, ≥ 70 according to the literature [29] and plots of the estimated restricted cubic spline function for age. Variables with < 10% missing values and with p < 0.20 on univariate analysis were tested in multivariate analyses. The variable selection involved using a stepwise model selection based on Akaike information criteria. Adjusted odds ratios with 95% CIs are given. The statistical significance threshold was set at p < 0.05. All analyses were performed by a dedicated statistician who used R 4.4.

## Results

### Asthma prevalence among hospitalized patients

**Fig. 1:**
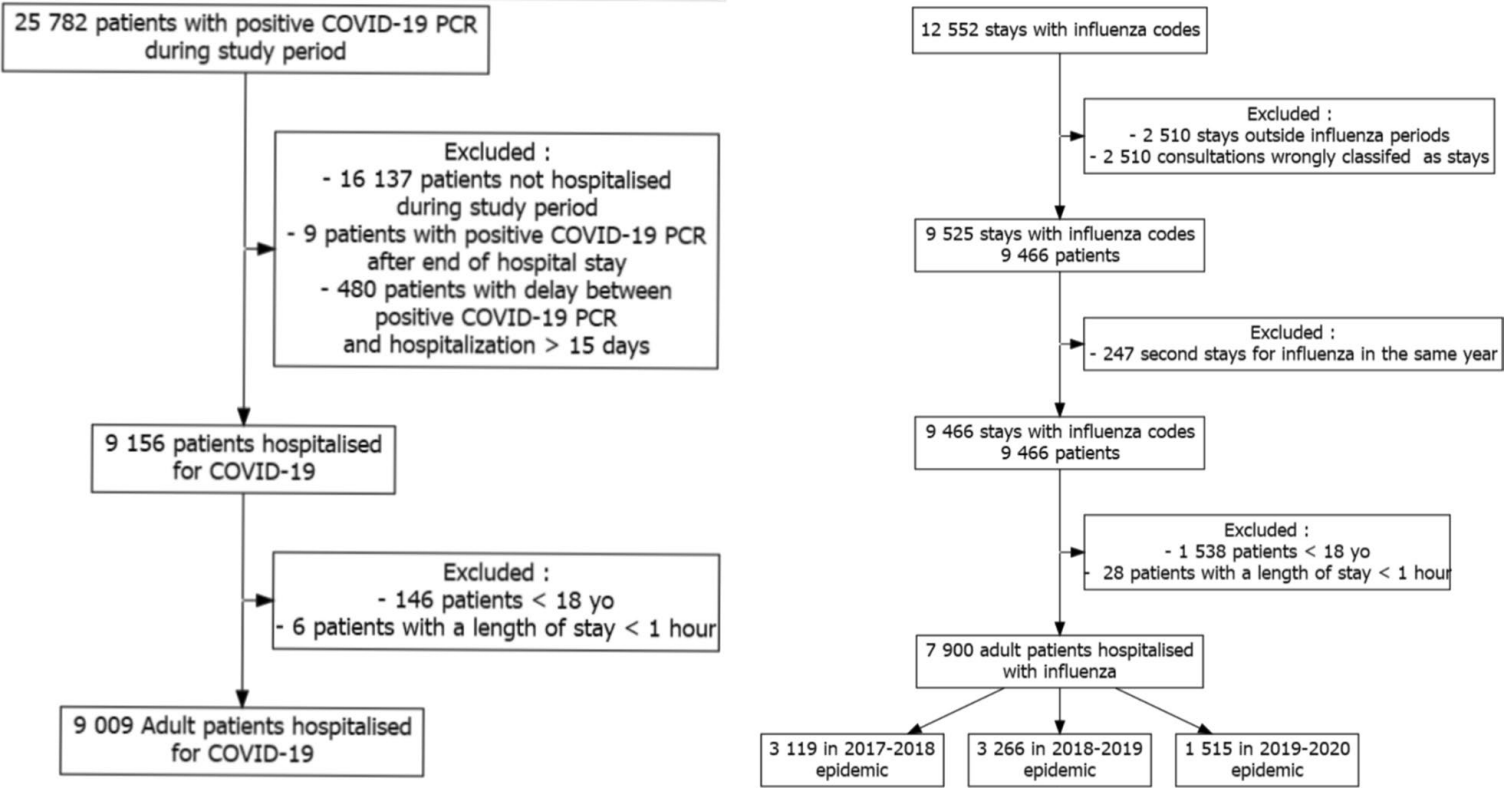
Flowcharts of COVID-19 and influenza patients

Between January 1 and July 3, 2020, 25,782 patients with a positive SARS-CoV-2 nasal PCR test were retrieved; 9009 were hospitalized (Fig. 1). During the 2017–2018 influenza season, 3119 patients were hospitalized and 3266 during the 2018–2019 influenza season (Fig. 1). Asthma prevalence was significantly higher for influenza than COVID-19 patients (9.1%, 95% CI [8.1–10.1] and 9.5%, 95% CI [8.5–10.5] for the 2017–2018 and 2018–2019 seasons, respectively, vs 4.5%, 95% CI [4.0–4.9], p < 0.001, for both comparisons).

### Characteristics of asthma patients

Detailed asthma patients characteristics are in Table 1. Among asthma patients, those with COVID-19 differed slightly from those with influenza during the 2018–2019 season: they were more frequently male, were older and had higher body-mass index (BMI). Differences were less pronounced for the 2017–2018 season. Surprisingly, the prevalence of heart failure or ischemic heart disease was lower in COVID-19 than influenza patients with asthma.

**Table 1:** Asthma patients characteristics at hospital admission by disease

	COVID-19 patients n = 402	2018–2019 influenza period patients n = 309	COVID-19 patients vs 2018–2019 influenza period patients p value	2017–2018 influenza period patients n = 283	COVID-19 patients vs 2017–2018 influenza period patients p value
Age (years)	68 [54–80]	66 [46–79]	0.045	67 [52–81]	0.47
Age ≥ 70	207 (51)	149 (48)	0.39	140 (49)	0.60
Male (%)	167 (42)	96 (31)	0.0048	107 (38)	0.34
Diabetes (%)	108 (27)	79 (26)	0.69	66 (23)	0.29
Obesity (%)	158 (39)	95 (31)	0.022	90 (32)	0.053
BMI (kg/m^2^)	28 [23–32]	26 [21–31]	0.0082	26 [22–31]	0.035
Missing	70	67		67	
High blood pressure (%)	190 (47)	144 (47)	0.88	139 (49)	0.64
Smoking (%)	217 (54)	167 (54)	1	158 (56)	0.64
Ischemic heart disease (%)	64 (16)	69 (22)	0.033	63 (22)	0.037
Heart failure (%)	65 (16)	85 (28)	< 0.001	82 (29)	< 0.001
Stroke (%)	31 (7.7)	27 (8.7)	0.68	24 (8.5)	0.78
Chronic renal failure (%)	53 (13)	54 (17)	0.11	52 (18)	0.068
Chronic renal failure on dialysis (%)	6 (1.5)	12 (3.9)	0.054	11 (3.9)	0.078

Data are median [IQR] or n (%)

Inhaled corticosteroids treatment was identified in hospital discharge reports for 56% of asthma patients during COVID-19-related hospitalization, 62% and 53% during the 2018–2019 and 2017–2018 influenza periods, respectively.

Among the 402 COVID-19 patients with asthma, 27 (6.7%) were hospitalized 1 month before, with asthma coded as the leading cause of admission or as an associated diagnosis. These rates were 17/309 (5.5%) and 16/283 (5.7%) for the 2018–2019 and 2017–2018 influenza periods, respectively.

As compared with non-asthma patients, asthma patients were younger in both COVID-19 and influenza groups (Additional file 1: Table S2). Asthma patients also had a lower proportion of males and higher prevalence of obesity and smoking, whatever the disease.

### Hospital stay characteristics and risk factors for outcome

COVID-19 asthma patients required higher levels of care than hospitalized influenza asthma patients (Table 2) and when admitted to an ICU, required mechanical ventilation more frequently, with longer stays. In-hospital mortality was higher for COVID-19 than influenza patients with asthma.

**Table 2:** Hospital stay for asthma patients and outcome by disease

	COVID-19 patients n = 402	2018–2019 influenza period patients n = 309	COVID-19 patients vs 2018–2019 influenza period patients p value	2017–2018 influenza period patients n = 283	COVID-19 patients vs 2017–2018 influenza period patients p value
Total length of hospital stay (days)	14 [7–55]	7 [4–13]	< 0.001	8 [5–15]	< 0.001
ICU	125 (31)	70 (23)	0.014	59 (21)	0.0028
Length of ICU stay (days)	10 [4–25]	4 [2–9]	< 0.001	4 [2–9]	< 0.001
Non-invasive ventilation	74 (18)	64 (21)	0.45	65 (23)	0.15
In ICU	71/125 (57)	50/70 (71)	0.043	50/59 (85)	< 0.001
Invasive ventilation	63 (16)	18 (5.8)	< 0.001	18 (6.4)	< 0.001
In ICU	63/125 (50)	18/70 (26)	< 0.001	17/59 (29)	0.0058
In-hospital death	73 (18)	9 (2.9)	< 0.001	12 (4.2)	< 0.001

Data are median [IQR] or n (%)

During the COVID-19 and influenza periods, asthma patients were more frequently hospitalized in an ICU than were non-asthma patients (Additional file 1: Table S3) but had similar requirements for invasive ventilation. Mortality was lower for all asthma patients groups than non-asthma patients, whatever the disease responsible for hospitalization.

Among asthma patients with COVID-19, smoking was a risk factor for admission to an ICU or death (Table 3, aOR = 1.57, 95% CI [1.03–2.40]), as was obesity (aOR = 1.55, 95% CI [1.00–2.41]). The odds were reduced with age ≥ 70 and female sex.

**Table 3:** Risk factors for admission to an ICU or hospital death for asthma patients by disease

	COVID-19 asthma patients n = 402 (170 events)			2018–2019 influenza period asthma patients n = 309 (74 events)			2017–2018 influenza period asthma patients n = 283 (63 events)		
	OR [95% CI]	aOR [95% CI]	p value	OR [95% CI]	aOR [95% CI]	p value	OR [95% CI]	aOR [95% CI]	p value
Age ≥ 70 years	0.60 [0.40–0.89]	0.64 [0.42–0.97]	0.037	0.66 [0.39–1.12]	0.49 [0.25–0.91]	0.033	0.84 [0.48–1.47]		
Female	0.39 [0.26–0.58]	0.38 [0.25–0.58]	< 0.001	0.72 [0.42 –1.26]			0.70 [0.40–1.24]		
Diabetes	1.02 [0.65–1.59]			1.57 [0.88–2.77]			1.16 [0.59–2.18]		
Obesity	1.48 [0.99–2.22]	1.55 [1.00–2.41]	0.049	1.11 [0.63–1.93]			2.22 [1.25–3.96]	2.22 [1.25–3.96]	0.0070
Smoking	1.73 [1.16–2.60]	1.57 [1.03–2.40]	0.035	2.92 [1.67–5.27]	2.95 [1.67–5.39]	< 0.001	1.38 [0.78–2.47]		
Ischemic heart disease	1.25 [0.73–2.13]			1.16 [0.62–2.12]			1.25 [0.64–2.37]		
Heart failure	1.12 [0.65–1.91]			1.49 [0.84–2.60]	2.05 [1.03–4.15]	0.040	1.72 [0.94–3.09]		
Stroke	1.13 [0.54–2.37]			0.70 [0.23–1.79]			1.49 [0.55–3.65]		
Chronic renal failure	1.15 [0.64–2.06]			0.78 [0.36–1.56]			1.55 [0.77–3.01]		

Inhaled corticosteroids (identified in hospital discharge reports) was not associated with poor outcome in the sensitivity univariate analysis (OR = 0.78, 95% CI [0.52–1.16]).

Obesity was associated with the ICU stay or hospital death during influenza infection for only the 2017–2018 group (Table 3, aOR = 2.22, 95% CI [1.25–3.96]) and smoking for only the 2018–2019 group (aOR = 2.95, 95% CI [1.67–5.39]). Other comorbid conditions such as diabetes, chronic renal failure or ischemic heart disease were not associated with poor outcomes, for both the COVID-19 and influenza groups. The association of inhaled corticosteroids and poor outcome differed by period: OR = 2.26, 95% CI [1.28–4.18] and OR = 1.14, 95% CI [0.65–2.01] for the 2018–2019 and 2017–2018 periods, respectively.

Separate analysis for admission to ICU or hospital death are presented in Additional file 1: Tables S8 and S9.

## Discussion

In this large multicenter retrospective study, including adults with PCR-confirmed SARS-CoV-2 infection, the prevalence of asthma patients requiring hospitalization for COVID-19 was significantly lower than that observed during the two influenza seasons, 2017–2018 and 2018–2019 (4.5% vs 9.1%). However, the prognosis was poorer with COVID-19 than influenza, with higher mortality rate, higher requirement for mechanical ventilation and longer hospital stay.

The asthma prevalence among patients hospitalized for COVID-19 varies widely among studies, from 0% in China (self-reported asthma) [30], 10.4% in the United Kingdom [31], to 10.7% in the United States [32], with asthma defined according to medical files. In the present study, the asthma prevalence was 4.5%, but the prevalence in the adult population in France is 7.4% [33]. Our results are in line with those published by Beltramo et al. using the same ICD-10 code-based diagnosis: the authors reported an asthma prevalence of 3.66% among patients hospitalized with COVID-19 in France and 4.9% among those hospitalized during the 2018–2019 influenza season [15]. With health care resource utilization and the consumption of 9 million insured German people and with similar methodology, asthma prevalence among COVID-19 hospitalized patients was 12% versus 15% in patients hospitalized for influenza during 2017–2019 [34].

The reasons for this lower representation of asthma patients with COVID-19 than influenza are still unclear [35]: suggested explanations are the role of strong adherence to protective shields [17, 18] and to treatments [19]; lower exposure to other respiratory viruses, especially influenza [36], allergens or pollutants during lockdown periods, as well as the potential effect of inhaled steroids on virus replication [37, 38] and decreased expression of the angiotensin-converting enzyme 2 receptor on asthma airway epithelial cells in patients with T2 phenotype [39, 40]. To distinguish the role of mechanical barriers from an intrinsic protection related to asthma and/or its treatments, comparing the prevalence of asthma among hospitalized patients during the successive waves of COVID-19, especially with new variants and lightened restrictions, would be useful.

Influenza and COVID-19 both trigger inflammatory processes that may exacerbate underlying health conditions or trigger cardiovascular events [41, 42] but induce different host responses. However, the higher respiratory pathogenicity of SARS-CoV-2 than the influenza virus was demonstrated in a nationwide cohort [43], with higher proportions of respiratory complications and requirement for mechanical ventilation (71% vs 61% among all patients in the ICU) and a three-fold increase in mortality, even in young people [44]. In our study, the need for invasive ventilation was 50% in the asthma population during COVID-19 as compared with 26% and 29% during influenza seasons. However, the mechanical ventilation rate may be difficult to read in COVID-19 patients because medical habits regarding ventilation and ICU access have changed a lot during the pandemic [45]. In our cohort, the in-hospital mortality in asthma patients was 18% for those with COVID-19 and 2.9% and 4.2% for the two influenza periods, with the two groups only slightly differing. Our study compared three groups of asthma patients hospitalized within the same care structures, which led to a more valid comparison [46].

The role of age and comorbidities (diabetes, cardiovascular diseases, obesity) was raised early as strong risk factors for COVID-19 and for severe disease [27, 47]. Surprisingly, in our cohort of asthma patients hospitalized with COVID-19, obesity and male sex were the main independent risk factors for admission to an ICU or death; diabetes and cardiovascular diseases were not associated with outcomes when adjusted on smoking. However, we did not include high blood pressure codes in the models because of the known risk of under-coding hypertension [48]. Obesity is a multifactorial disorder with multiple effects on the host response to infection and leads to a heightened pro-inflammatory response and blunted anti-viral response, which explains the increased virus-induced disease severity with obesity in both influenza and COVID-19 [49]. Indeed, the prevalence of obesity in critically ill COVID-19 patients in another French study was higher, by 2.88 (95% CI 2.19–3.66), than in the French general population after standardization on age and sex [50]. Obesity (BMI ≥ 30 kg/m^2^) has been widely described as an main independent risk factor of poor outcome [51]. Our estimated OR was close to that of other studies, which suggests that asthma does not increase the risk with obesity, although we cannot exclude classification bias in our study regarding the use of word “obesity” in medical reports. For asthma patients, admission to an ICU or death was not significantly associated with obesity for the 2018–2019 influenza period but was for the 2017–2018 period. Such contrasting findings have also been observed with the influenza A pandemic H1N1 [52], seasonal A(H1N1)pdm09 influenza [53, 54] or with other influenza periods [53, 55].

Age ≥ 70 years was significantly associated with less frequent admission to an ICU or death for asthma patients in our study for COVID-19, which can be contradictory with previous data [29, 56]. Limitation of transfer to an ICU for ethical reasons in a context of overburdening hospital care in our study may account for this discrepancy, since 25% of our asthma population was > 80 years old. Indeed, life-sustaining treatments to avoid unreasonable aggressive treatments increases with age and number of comorbid conditions before critical illness [57].

Age ≥ 70 years was significantly associated with less frequent admission to an ICU or death during the 2018–2019 period, as in a Spanish study [53]. We performed sensitivity analysis with hospital death as outcome: age ≥ 70 was an independent risk factor for COVID-19. For influenza, age ≥ 70 was not significant in univariate analysis but it could be explained by low statistical power, as only 9 and 12 deaths were observed for both influenza periods.

This study has several limitations. The study period for COVID-19 included only the year 2020, before the SARS-CoV-2 vaccine campaigns and before the emergence of new viral variants. Influenza-related hospitalizations were defined only with ICD-10 codes because definite results for influenza from PCR analysis were not imported into the EDS. Moreover, before the COVID-19 pandemic, a PCR test for respiratory viral infection was not systematically performed in hospital settings. Therefore, we cannot exclude that some patients with other respiratory virus infections could be misclassified.

In the EDS, coding for epidemiological studies is ruled by economic criteria and not completeness. Because asthma and others comorbidities could be identified only with diagnostic discharge codes, some patients could have been mistakenly classified as without comorbidities if the diagnostic code have not been detected. This is particularly important regarding obesity: due to missing BMI values in the EDS, obesity was defined by natural language processing of medical charts, introducing potential classification bias, especially between overweight and obese categories. Moreover, we cannot exclude ICD-10 coding errors in our database. The association between severity and patients characteristics may be biased because of residual confounding, especially for influenza vaccination status [14] and environmental factors (air pollution, pollinating season etc.) associated with asthma exacerbations [35].

The most disappointing point is that we failed to strongly characterize asthma control or severity; especially, treatments were identified across the EDS database by natural language processing of discharge reports, which probably explains the low proportion of patients identified with inhaled steroids treatment. Also, we could not assess whether the drugs were prescribed before (admission treatment) or during hospitalization. However, Choi et al. found no significant association between previous asthma medication (inhaled corticosteroids alone or long-acting beta2-agonists) and ICU admission for COVID-19 among asthmatic patients [58]. Adir et al*.* reported a higher risk of moderate to severe COVID-19 or all-cause mortality within 90 days after a positive PCR test result for only patients with at least three prescriptions of steroids in the previous year [26]. Also, the findings on seasonal influenza cannot necessarily be extrapolated to other seasonal influenza, because of potentially other strains.

## Conclusions

In conclusion, this study finds a lower representation of asthma patients among those hospitalized for COVID-19 and a negative impact of obesity on outcome. However, as shown in the general population [43], outcomes were poorer for COVID-19 than influenza patients, both with asthma, for reasons that remain to be elucidated. These data highlight the importance of protective shields and vaccination against influenza and SARS-CoV-2 in this population.

## Supplementary Information

**Additional file 1: Figure S1.** Distribution of International Statistical Classification of Diseases, Tenth Revision (ICD-10) codes. **Table S1.** List of ICD-10 codes. **Table S2.** Characteristics of patients at admission to an AP-HP hospital for COVID-19 or influenza by presence or not of asthma antecedent. **Table S3.** Hospital stays and outcome for patients hospitalized in an AP-HP hospital for COVID-19 or influenza by presence or not of asthma antecedent. **Table S4.** Characteristics of patients at admission to an AP-HP hospital for influenza in 2019–2020 by presence or not of asthma antecedent. **Table S5.** Characteristics of asthma patients at hospital admission in 2019–2020 by infectious disease: COVID-19 or influenza. **Table S6.** Hospital stays and outcome for asthma patients at hospital admission in 2019–2020 by infectious disease: COVID-19 or 2019–2020 influenza. **Table S7.** Hospital stays and outcome for patients hospitalized in an AP-HP hospital for influenza in 2019–2020 by presence or not of asthma antecedent. **Table S8.** Risk factors for admission to an ICU for asthma patients by disease. **Table S9.** Risk factors for hospital death for asthma patients by disease.

## Data Availability

The data that support the findings of this study are available from AP-HP EDS but are not publicly available due to French Data Protection Act. After EDS ethics committee authorization, data is only accessible on a personnel “project space” on a secured platform and we process the data on this platform without being able to retrieve it. Data are however available upon reasonable request from AP-HP EDS and with permission ethics committee of EDS.

## Abbreviations

aORs: Adjusted odds-ratio
BMI: Body Mass Index
CI: Confidence intervals
COVID-19: Coronavirus disease 19
ICU: Intensive Care Unit
ICD-10: International Statistical Classification of Diseases, Tenth Revision
OR: Odds ratio
SARS-CoV-2: Severe acute respiratory syndrome coronavirus 2
