# Supplementary material for: Outcomes and risk factors with COVID-19 or influenza in hospitalized asthma patients
Source: Respir Res. 2022 Dec 13;23:342. doi: 10.1186/s12931-022-02265-6 (PMC9745693; doi:10.1186/s12931-022-02265-6)
Supplement: Supplementary file 1 — Additional file 1: Figure S1. Distribution of International Statistical Classification of Diseases, Tenth Revision (ICD-10) codes. Table S1. List of ICD-10 codes. Table S2. Characteristics of patients at admission to an AP-HP hospital for COVID-19 or influenza by presence or not of asthma antecedent. Table S3. Hospital stays and outcome for patients hospitalized in an AP-HP hospital for COVID-19 or influenza by presence or not of asthma antecedent. Table S4. Characteristics of patients at admission to an AP-HP hospital for influenza in 2019–2020 by presence or not of asthma antecedent. Table S5. Characteristics of asthma patients at hospital admission in 2019–2020 by infectious disease: COVID-19 or influenza. Table S6. Hospital stays and outcome for asthma patients at hospital admission in 2019–2020 by infectious disease: COVID-19 or 2019–2020 influenza. Table S7. Hospital stays and outcome for patients hospitalized in an AP-HP hospital for influenza in 2019–2020 by presence or not of asthma antecedent. Table S8. Risk factors for admission to an ICU for asthma patients by disease. Table S9. Risk factors for hospital death for asthma patients by disease. [file 12931_2022_2265_MOESM1_ESM.docx]

**Additional file 1.**

Figure S1: Distribution of International Statistical Classification of Diseases, Tenth Revision (ICD-10) codes.


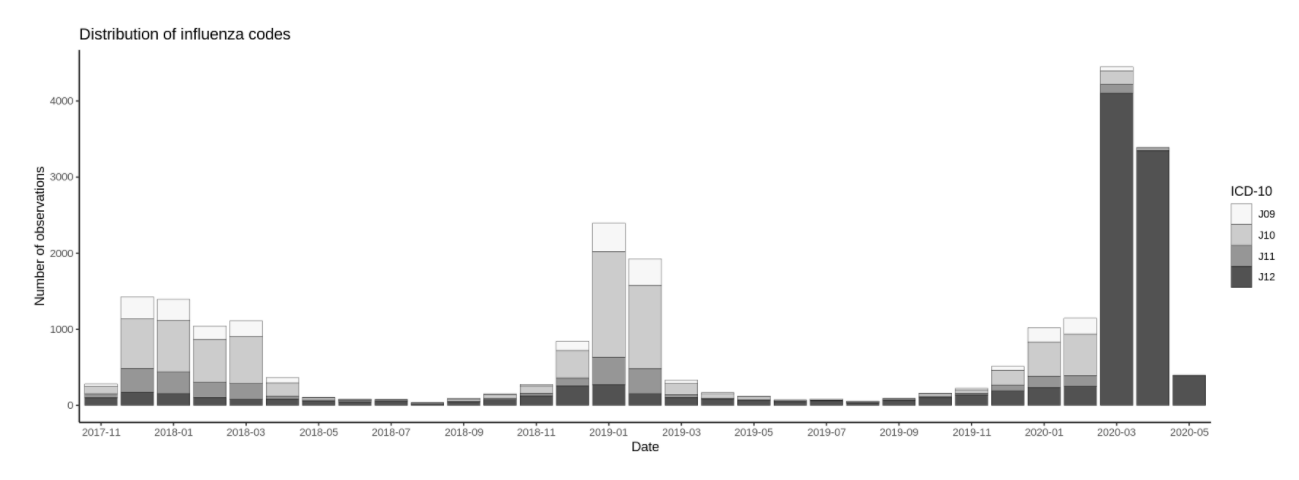


Table S1: List of ICD-10 codes.

| ICD-10 codes | Disease |
| --- | --- |
| J45 | Asthma |
| J46 | Status asthmaticus |
| J09 | Influenza due to certain identified influenza virus |
| J10 | Influenza due to other identified influenza virus |
| J11 | Influenza, virus not identified |
| E10,E11,E12,E13,E14,R739 | Diabetes |
| E66 | Obesity |
| I20, I21,I22,I23,I24,I25,Z95 | Ischemic heart disease |
| I50 | Heart failure |
| I60, I61, I62, I63, I64, I69, Z8670 | Stroke |
| F17 | Use of tobacco |
| N18, N19 | Chronic renal failure |
| N185 | Chronic Renal failure on dialysis |
| I10, I11, I12, I13,I15 | High blood pressure |

Table S2: Characteristics of patients at admission to an AP-HP hospital for COVID-19 or influenza by presence or not of asthma antecedent

| Covid-19 patients | Total  n=9 009 | Asthma patients  n=402 | Non-asthma patients  n=8 607 | p |
| --- | --- | --- | --- | --- |
| Age (years) | 70 [57-83] | 68 [54-80] | 71 [57-83] | 0.0017 |
| Male (%) | 5 189 (58) | 167 (42) | 5 022 (58) | **<0.001** |
| Diabetes (%) | 2 514 (28) | 108 (27) | 2 406 (28) | 0.69 |
| Obesity (%) | 2 348 (26) | 158 (39) | 2 190 (25) | **<0.001** |
| BMI (kg/m2)  missing | 26 [22-30]  2 549 | 28 [23-32]  70 | 26 [22-30]  2 479 | **<0.001** |
| High blood pressure (%) | 4 325 (48) | 190 (47) | 4 135 (48) | 0.80 |
| Smoking (%) | 4 346 (48) | 217 (54) | 4 129 (48) | 0.019 |
| Ischemic heart disease (%) | 1 527 (17) | 64 (16) | 1 483 (17) | 0.63 |
| Heart failure (%) | 1 294 (14) | 65 (16) | 1 229 (14) | 0.31 |
| Stroke (%) | 887 (9.8) | 31 (7.7) | 856 (9.9) | 0.17 |
| Chronic renal failure (%) | 1 564 (17) | 52 (13) | 1 512 (18) | 0.015 |
| Chronic renal failure on dialysis (%) | 299 (3.3) | 6 (1.5) | 293 (3.4) | 0.032 |
| 2018-2019 influenza period | Total  n=3 266 | Asthma patients  n=309 | Non-asthma patients  n=2 597 | p |
| Age (years) | 74 [59-85] | 66 [46-79] | 75 [60-86] | **<0.001** |
| Male (%) | 1 544 (47) | 96 (31) | 1 448 (49) | **<0.001** |
| Diabetes (%) | 843 (26) | 79 (26) | 764 (28) | 0.032 |
| Obesity (%) | 619 (19) | 95 (31) | 524 (18) | **<0.001** |
| BMI ( kg/m2) Missing | 24[21-29] 1 068 | 26 [21-31]  67 | 24 [21-28]  952 | 0.0043 |
| High blood pressure (%) | 1 657 (51) | 144 (47) | 1 513 (51) | 0.13 |
| Smoking (%) | 1600 (49) | 167 (54) | 1 433 (48) | 0.064 |
| Ischemic heart disease (%) | 838 (26) | 69 (22) | 769 (26) | 0.17 |
| Heart failure (%) | 991 (30) | 85 (28) | 906 (31) | 0.27 |
| Stroke (%) | 360 (11) | 27 (8.7) | 333 (11) | 0.21 |
| Chronic renal failure (%) | 756 (23) | 54 (17) | 702 (24) | 0.013 |
| Chronic renal failure on dialysis (%) | 144 (4.4) | 12 (3.9) | 132 (4.5) | 0.77 |
| 2017-2018 influenza period | Total  n=3 119 | Asthma patients  n=283 | Non-asthma patients  n=2 836 | p |
| Age (years) | 72 [58-84] | 67 [52-81] | 73 [58-84] | **<0.001** |
| Male (%) | 1466 (47) | 107 (38) | 1 359 (48) | 0.0012 |
| Diabetes (%) | 780 (25) | 66 (23) | 714 (25) | 0.52 |
| Obesity (%) | 643 (21) | 90 (32) | 553 (19) | **<0.001** |
| BMI (kg/m2) Missing | 25 [21-29]  1 068 | 26 [22-31]  67 | 24 [21-29]  1 001 | 0.0022 |
| High blood pressure (%) | 1 547 (50) | 139 (49) | 1 408(50) | 0.90 |
| Smoking (%) | 1 551 (50) | 158 (56) | 1 393 (49) | 0.034 |
| Ischemic heart disease (%) | 772 (25) | 63 (22) | 709 (25) | 0.35 |
| Heart failure (%) | 8885 (28) | 82 (29) | 803 (28) | 0.84 |
| Stroke (%) | 394 (13) | 24 (8.5) | 370 (13) | 0.030 |
| Chronic renal failure (%) | 663 (21) | 49 (17) | 614 (22) | 0.094 |
| Chronic renal failure on dialysis (%) | 161 (5.2) | 11 (3.9) | 150 (5.3) | 0.40 |

Data are median [IQR] or n (%); BMI, body mass index

Table S3: Hospital stays and outcome for patients hospitalized in an AP-HP hospital for COVID-19 or influenza by presence or not of asthma antecedent

| COVID-19 patients | Total  n=9 009 | Asthma patients  n=402 | Non-asthma patients  n=8 607 | p |
| --- | --- | --- | --- | --- |
| Total length of stay (days) | 13 [6-49] | 14 [7-55] | 13 [6-48] | 0.15 |
| ICU  - length of stay in ICU (days) | 2 315 (26)  10 [4-22] | 125 (31) 10 [4-25] | 2 190 (25) 10 [4-21] | 0.014  0.93 |
| Non-invasive ventilation - in ICU | 1 384 (15) 1 257/2 315 (54) | 74 (18) 71/125 (57) | 1 310 (15) 1 186/2 190 (54) | 0.089  0.58 |
| Invasive ventilation  - in ICU | 1 186 (13) 1 183/2 315 (51) | 63 (16) 63/125 (50) | 1 123 (13) 1 120/2 190 (51) | 0.13  0.93 |
| In-hospital death | 2 125 (24) | 73 (18) | 2 052 (24) | 0.0076 |
| 2018-2019 influenza period | Total  n=3 266 | Asthma patients n=309 | Non-asthma patients  n=2 957 | p |
| Total length of stay (days) | 8 [4-18] | 7 [4-13] | 8 [4-19] | 0.005 |
| ICU  - length of stay in ICU (days) | 554 (17) 4 [2-9] | 70 (23)  4 [2-9] | 484 (16) 4 [2-9] | 0.0067  0.91 |
| Non-invasive ventilation   - In ICU | 546 (17) 404/554 (73) | 64 (21) 50/70 (71) | 482 (16) 354/484 (73) | 0.054  0.77 |
| Invasive ventilation   - In ICU | 198 (6.1)  182/554 (33) | 18 (5.8) 18/70 (26) | 180 (6.1) 164/484 (34) | 1 0.22 |
| In-hospital death | 210 (6.4) | 9 (2.9) | 201 (6.8) | 0.0068 |
| 2017-2018 influenza period | Total  n=3 119 | Asthma patients  n=283 | Non-asthma patients  n=2 836 | p |
| Total length of stay (days) | 8 [4-17] | 8 [5-15] | 8 [4-17] | 0.84 |
| ICU  - length of stay in ICU (days) | 552 (18) 4 [2-9] | 59 (21) 4 [2-9] | 493 (17) 4 [2-9] | 0.16 0.74 |
| Non-invasive ventilation   - In ICU | 604 (19)  416/552 (75) | 65 (23)  50/59 (85) | 539 (19)  366/493 (74) | 0.11  0.080 |
| Invasive ventilation   - In ICU | 230 (7.4) 208/552 (38) | 18 (6.4) 17/59 (29) | 212 (7,5) 191/493 (39) | 0.55  0.16 |
| In-hospital death | 214 (6.9) | 12 (4.2) | 202 (7.1) | 0.083 |

Data are median [IQR] or n (% ); ICU, intensive care unit

Table S4: Characteristics of patients at admission to an AP-HP hospital for influenza in 2019-2020 by presence or not of asthma antecedent

|  | Total  n=1 515 | Asthma patients  n=155 | Non-asthma patients  n=1 360 | p |
| --- | --- | --- | --- | --- |
| Age (years) | 63 [45-78] | 59 [43-72] | 64 [45-78] | 0.026 |
| Male (%) | 752 (50) | 52 (34) | 700 (51) | **<0.001** |
| Diabetes (%) | 322 (21) | 37 (24) | 285 (21) | 0.41 |
| Obesity (%) | 284 (19) | 55 (35) | 229 (17) | **<0.001** |
| BMI (kg/m2)  missing | 25 [21-29] 498 | 27 [23-32]  35 | 24 [21-28] 463 | **<0.001** |
| High blood pressure (%) | 568 (37) | 63 (41) | 505 (37) | 0.43 |
| Smoking (%) | 711 (47) | 75 (48) | 636 (47) | 0.73 |
| Ischemic heart disease (%) | 259 (17) | 30 (19) | 229 (17) | 0.43 |
| Heart failure (%) | 305 (20) | 30 (19) | 275 (20) | 0.92 |
| Stroke (%) | 127 (8.4) | 7 (4.5) | 120 (8.8) | 0.067 |
| Chronic renal failure (%) | 240 (16) | 19 (12) | 221 (16) | 0.24 |
| Chronic renal failure on dialysis (%) | 70 (4.6) | 6 (3.9) | 64 (4.7) | 0.84 |

Data are median [IQR] or n (%); BMI, body mass index

Table S5: Characteristics of asthma patients at hospital admission in 2019-2020 by infectious disease: COVID-19 or influenza

|  | COVID-19 patients  n=402 | 2019-2020 influenza period patients  n=155 | p |
| --- | --- | --- | --- |
| Age (years) | 68 [54-80] | 59 [43-72] | **< 0.001** |
| Male (%) | 167 (42) | 52 (34) | 0.10 |
| Diabetes (%) | 108 (27) | 37 (24) | 0.52 |
| Obesity (%) | 158 (39) | 55 (35) | 0.44 |
| BMI (kg/m2)  Missing | 28 [23-32]  70 | 27 [23-32]  35 | 0.65 |
| High blood pressure (%) | 190 (47) | 63 (41) | 0.18 |
| Smoking (%) | 217 (54) | 75 (48) | 0.64 |
| Ischemic heart disease (%) | 64 (16) | 30 (19) | 0.38 |
| Heart failure (%) | 65 (16) | 30 (19) | 0.38 |
| Stroke (%) | 31 (7.7) | 7 (4.5) | 0.26 |
| Chronic renal failure (%) | 53 (13) | 21 (14) | 0.89 |
| Chronic renal failure on dialysis (%) | 6 (1.5) | 6 (3.9) | 0.10 |

Data are median [IQR] or n (%); BMI, body mass index

Table S6: Hospital stays and outcome for asthma patients at hospital admission in 2019-2020 by infectious disease: COVID-19 or 2019-2020 influenza

|  | COVID-19 patients n=402 | 2019-2020 influenza period patients  n=155 | p |
| --- | --- | --- | --- |
| Total length of stay (days) | 14 [7-55] | 6 [3-12] | **<0.001** |
| ICU:  -length of stay in ICU (days) | 125 (31)  10 [4-25] | 39 (25)  4 [1-5] | 0.18  **<0.001** |
| Non-invasive ventilation   - In ICU | 74 (18)  71/125 (57) | 37 (24)  27/39 (69) | 0.16  0.19 |
| Invasive ventilation   - In ICU | 63 (16) 63/125 (50) | 7 (4.5)  7/39 (18) | **<0.001 <0.001** |
| In-hospital death | 73 (18) | 8 (5.2) | **<0.001** |

Data are median [IQR] or n (%); ICU, intensive care unit

Table S7: Hospital stays and outcome for patients hospitalized in an AP-HP hospital for influenza in 2019-2020 by presence or not of asthma antecedent

|  | Total  n=1 515 | Asthma patients  n=155 | Non-asthma patients  n=1 360 | p |
| --- | --- | --- | --- | --- |
| Total length of stay (days) | 6 [2-14] | 6 [3-12] | 7 [2-14] | 0.61 |
| ICU:  - length of stay in ICU (days) | 294(19)  4 [1-9] | 39 (25)  4 [1-5] | 255 (19)  4 [1-10] | 0.068 0.061 |
| Non-invasive ventilation   - In ICU | 261 (17)  199 / 294 (68) | 37 (24)  27 / 39 (69) | 224 (16)  172 / 255 (67) | 0.025  1 |
| Invasive ventilation   - In ICU | 115 (7.6)  106 / 294 (36) | 7 (4.5)  7 / 39 (18) | 108 (7.9)  99 / 255 (39) | 0.15  0.012 |
| In-hospital death | 79 (5.2) | 8 (5.2) | 71 (5.2) | 1 |

Data are median [IQR] or n (%); ICU, intensive care unit

Table S8: Risk factors for admission to an ICU for asthma patients by disease

| **ICU** | **COVID-19 asthma patients n=402  (125 events)** | | | **2018-2019 influenza asthma patients n=309 (70 events)** | | | | **2017-2018 influenza ashma patients n=283 (60 events)** | | | |
| --- | --- | --- | --- | --- | --- | --- | --- | --- | --- | --- | --- |
|  | **OR  [95% CI]** | **aOR [95% CI]** | **p value** | **OR [95% CI]** | **aOR  [95% CI]** | **p value** | **OR [95% CI]** | | **aOR [95% CI]** | **p value** |  |
| Age ≥ 70 yo | 0.23  [0.14-0.37] | 0.26  [0.15-0.42] | **<0.001** | 0.45  [0.25-0.79] | 0.42  [0.22-0.75] | 0.0043 | 0.46  [0.25-0.83] | | 0.45  [0.24- 0.82] | 0.010 |  |
| Female Patients | 0.47  [0.30-0.72] | 0.42  [0.26-0.67] | **<0.001** | 0.65  [0.37-1.14] |  |  | 0.63  [0.35-1.12] | | 0.58  [0.31-1.08] | 0.086 |  |
| Diabetes | 0.91  [0.56-1.46] |  |  | 1.75  [0.97-3.10] | 2.11  [1.13-3.95] | 0.019 | 1.26  [0.64-2.38] | |  |  |  |
| Obesity | 2.48  [1.61-3.83] | 2.28  [1.41-3.71] | **<0.001** | 1.23  [0.69-2.16] |  |  | 2.08  [1.15-3.74] | | 2.25  [1.22-4.17] | 0.0094 |  |
| Smoking | 1.57  [1.02-2.42] | 1.53  [0.96-2.46] | 0.078 | 2.61  [1.48-4.73] | 2.58  [1.45-4.73] | 0.0016 | 1.62  [0.91-2.97] | | 1.35  [0.73- 2.56] | 0.34 |  |
| Ischemic heart disease | 0.70  [0.37-1.26] |  |  | 1.28  [0.67-2.34] |  |  | 1.36  [0.69-2.59] | |  |  |  |
| Heart failure | 0.56  [0.29-1.03] |  |  | 1.28  [0.71-2.27] |  |  | 1.30 [0.69-2.37] | |  |  |  |
| Stroke | 0.90  [0.38-1.95] |  |  | 0.76  [0.25-1.94] |  |  | 1.60  [0.59- 3.92] | |  |  |  |
| Chronic Renal failure | 0.61  [0.30-1.17] |  |  | 0.85  [0.40-1.70] |  |  | 1.14  [0.54- 2.29] | |  |  |  |

Table S9: Risk factors for hospital death for asthma patients by disease

| **In hospital -death** | **COVID-19 asthma patients n=402 (73 deaths)** | | | **2018-2019 influenza asthma patients n=309 (9 deaths)** | | **2017-2018 influenza asthma patients n=283 (12 deaths)** | |
| --- | --- | --- | --- | --- | --- | --- | --- |
|  | **OR [95% CI]** | **Adjusted OR  [95% CI]** | **p value** | **OR [95% CI]** | **p value** | **OR [95% CI]** | **p value** |
| Age ≥ 70 yo | 3.17  [1.86-5.56] | 3.09  [1.78-5.52] | **<0.001** | 2.58  [0.67-12.4] | 0.17 | 2.41  [0.74-9.19] | 0.15 |
| Female Patients | 0.56  [0.33-0.92] | 0.50  [0.29- 0.85] | 0.010 | 1.60  [0.38-10.9] | 0.55 | 0.59  [0.18-1.95] | 0.38 |
| Diabetes | 1.03  [0.57-1.80] |  |  | 0.83  [0.12-3.51] | 0.81 | 1.69  [0.44-5.54] | 0.42 |
| Obesity | 0.61  [0.35-1.04] |  |  | - |  | 3.17   [0.98-11.0] | 0.053 |
| Smoking | 1.69  [1.01- 2.89] |  |  | 1.73  [0.45-8.30] | 0.44 | 0.55  [0.16-1.77] | 0.32 |
| Ischemic heart disease | 2.22  [1.19- 4.05] |  |  | 0.99  [0.15-4.23] | 1 | 1.17   [0.25- 4.07] | 0.82 |
| Heart failure | 1.96  [1.04- 3.59] |  |  | 3.14  [0.45-13.9] | 0.0014 | 3.66  [1.13-12.7] | 0.030 |
| Stroke | 1.35  [0.52-3.11] |  |  | 3.14  [0.45-13.9] | 0.21 | 3.97   [0.83- 14.5] | 0.079 |
| Chronic Renal failure | 2.47 [1.27-4.65] | 1.87  [0.94-3.63] | 0.067 | 3.28  [0.17-20.3] | 0.35 | 2.37   [0.12-14.2] | 0.47 |
